# Fitness variation in isogenic populations leads to a novel evolutionary mechanism for crossing fitness valleys

**DOI:** 10.1038/s42003-018-0160-1

**Published:** 2018-09-26

**Authors:** Debra Van Egeren, Thomas Madsen, Franziska Michor

**Affiliations:** 1 Department of Biostatistics and Computational Biology, Dana-Farber Cancer Institute, Boston, MA 02115 USA; 2 Department of Systems Biology, Harvard Medical School, Boston, MA 02115 USA; 3 Department of Biostatistics, Harvard T. H. Chan School of Public Health, Boston, MA 02215 USA; 4 Department of Stem Cell and Regenerative Biology, Harvard University, Cambridge, MA 02138 USA; 5 The Center for Cancer Evolution, Dana-Farber Cancer Institute, Boston, MA 02115 USA; 6 The Broad Institute of Harvard and MIT, Cambridge, MA 02139 USA; 7 The Ludwig Center at Harvard, Boston, MA 02115 USA

## Abstract

Individuals in a population often have different fitnesses even when they have identical genotypes, but the effect of this variation on the evolution of a population through complicated fitness landscapes is unknown. Here, we investigate how populations with non-genetic fitness variation cross fitness valleys, common barriers to adaptation in rugged fitness landscapes in which a population must pass through a deleterious intermediate to arrive at a final advantageous stage. We develop a stochastic computational model describing the dynamics of an asexually reproducing population crossing a fitness valley, in which individuals of the same evolutionary stage can have variable fitnesses. We find that fitness variation that persists over multiple generations increases the rate of valley crossing through a novel evolutionary mechanism different from previously characterized mechanisms such as stochastic tunneling. By reducing the strength of selection against deleterious intermediates, persistent fitness variation allows for faster adaptation through rugged fitness landscapes.

## Abstract (web summary)

Debra Van Egeren et al. present a stochastic computational model of the dynamics of an asexually reproducing population, such as somatic or cancer cells, crossing a fitness valley. They find that fitness variation persisting across generations promotes weaker selection against deleterious intermediates, thereby increasing the rate of valley crossing.

## Introduction

**Fig. 1:**
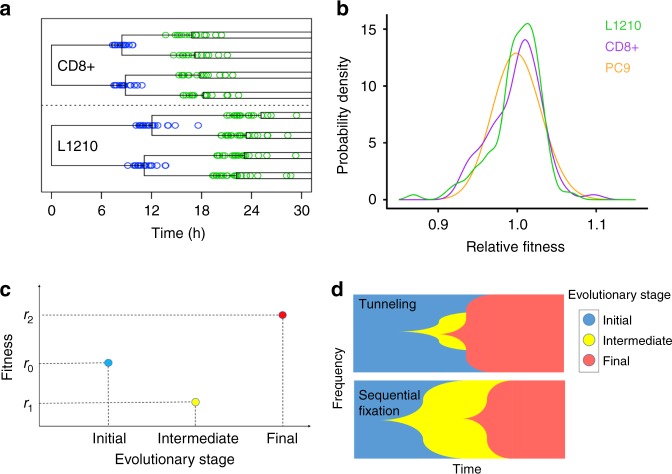
Variable fitness values in isogenic populations and mechanisms of adaptation to rugged fitness landscapes. **a** Single cell interdivision times in lineages of primary murine CD8+ T cells and a murine lymphocytic leukemia cell line (L1210). Division times of cells in each generation (green and blue circles) are variable, as shown by their distribution across the time axis. **b** Fitness distributions estimated from single cell PC9 (a human lung cancer cell line), CD8+, and L1210 interdivision time data. Fitness is estimated as the reciprocal of the interdivision time. All distributions are scaled to have mean 1 and equal variance. **c** Illustration of a fitness valley. Individuals of the initial evolutionary stage have fitness *r*_0_, individuals of the intermediate stage have a lower fitness *r*_1_, and individuals of the final stage have a higher fitness *r*_2_. **d** Illustration of mechanisms of fitness valley crossing. If stochastic tunneling occurs, individuals of the final evolutionary stage emerge before the individuals of the intermediate stage take over the population (top). Without tunneling, the disadvantageous intermediate type fixes before the final advantageous trait emerges and fixes in the population (bottom)

Variation in reproductive fitness among individuals of a population is common. In some cases, this variation is due to genetic heterogeneity, where multiple segregating mutations with different fitness effects exist simultaneously in the population^1,2^. However, recent work quantifying interdivision times in isogenic mammalian cell populations demonstrated that fitness variation can exist even among individuals with the same genotype^3,4^ (Fig. 1a, b). This non-genetic fitness variation may originate from several different intrinsic and extrinsic sources, each of which can have different persistence timescales. For example, gene expression heterogeneity has the potential to modify a cell’s fitness^5^ and is a source of non-genetic resistance in cancer^6^. These transcriptional fluctuations have been shown to persist over 3–4 human cell divisions^7^. Epigenetic modifications such as DNA methylation persist over longer timescales (>20 cell divisions)^8^ and may represent an additional source of stable fitness variation in genetically identical individuals. Here, we investigated how this non-genetic fitness variation affects evolution through complex fitness landscapes, and how these effects depend on the magnitude and persistence length of fitness variation.

Background fitness variation has previously been shown to affect evolutionary trajectories by reducing the strength of selection for or against new mutations. This phenomenon is known as clonal interference or the Hill–Robertson effect^9,10^ in the context of genetic fitness variation. When new mutations arise, they are linked to the genetic background of the individual in which the mutation occurred. If a population includes many different genotypes, this linkage leads to greater variability in the relative fitness of new mutants, reducing the average magnitude of the fitness effect of the mutation and leading to weaker selection^11–13^. In sexually reproducing populations, genetic recombination breaks the linkage between the mutation of interest and other loci in the original genotype, removing any fitness effect conferred by that genotype in subsequent generations^14^. While the Hill–Robertson effect has been extensively investigated in the context of a single locus or multiple independent loci, the effect of fitness variation on populations in rugged fitness landscapes with sign epistasis is less well understood. Here, we investigate how stable fitness variation affects the evolution of populations crossing fitness valleys, which are frequently found in complex fitness landscapes.

Fitness valleys are barriers to adaptation that exist when a population of individuals can acquire an advantageous trait only by passing through an intermediate stage of lower fitness (Fig. 1c). During tumorigenesis, for example, fitness valleys are represented by situations in which cells that lost one functional copy of a tumor suppressor gene are less fit than the original population^15^. Other examples arise during the development of antibiotic resistance in bacteria^16^, and immune system escape in influenza^17^ and HIV^18^, and affect the speed and trajectory of evolutionary adaptation in these populations. In particular, selection against the deleterious intermediate stage decreases the rate at which individuals of the final, advantageous evolutionary stage arise, rendering adaptation to the fitness peak difficult. Previous work^19^ characterizing the dynamics of valley crossing identified two mechanisms by which populations can overcome this adaptation barrier (Fig. 1d). First, in smaller populations with a weakly deleterious intermediate, the intermediate stage may reach 100% frequency via drift, after which final-stage individuals emerge and sweep throughout the population. This evolutionary mechanism is referred to as sequential fixation. However, increasing the population size or the valley depth decreases the chance of the population evolving along this route. In these cases, valley crossing often occurs through a second mechanism known as stochastic tunneling, in which final-stage individuals emerge and reach fixation in the population before the deleterious intermediate does^20–22^. The rates of both of these valley crossing mechanisms have been derived for asexually reproducing populations without background genetic variation^23^.

The dynamics of fitness valley crossing has previously been shown in models of viral dynamics to be affected by genetic clonal interference and recombination. Neher and Shraiman derived the rate of fixation of new mutations and the rate of stochastic tunneling for a mathematical model of HIV replication and recombination in which mutations are common and clonal interference is frequent^24^. They showed that the rate of fixation of the deleterious intermediate and the rate of stochastic tunneling are higher in populations with more clonal interference, i.e., low recombination rates and high background fitness variation, leading to an increased rate of fitness valley crossing. We hypothesized that a similar increase in valley crossing rates also results from non-genetic variation in genetically identical individuals. While prior theoretical work suggests that a similar reduction in selection strength results from noise in gene expression levels^25^, the effect of non-genetic background fitness on evolution in more complex fitness landscapes has not yet been systematically explored.

Here, we design a computational model in which small, frequent stochastic fitness alterations generate population-level fitness variation, recapitulating many of the features observed in isogenic cell populations. Using this model, we find that non-genetic fitness variation that persists across multiple generations increases the rate of valley crossing. This increase in the rate of valley crossing occurs via a new mechanism in which the emergence of individuals of the intermediate stage with high-fitness backgrounds increases the rate of intermediate-stage fixation. Additionally, we show that the adaptation rate is increased as the magnitude of the background non-genetic fitness variation increases or as the persistence timescale of the fitness alterations lengthens. Therefore, by reducing the efficacy of selection against weakly deleterious intermediate traits, stable background fitness variation increases the rate of adaptation in rugged fitness landscapes and leads to a novel evolutionary mechanism of crossing fitness valleys.

## Results

### Fitness alterations lead to steady-state fitness variation

**Fig. 2:**
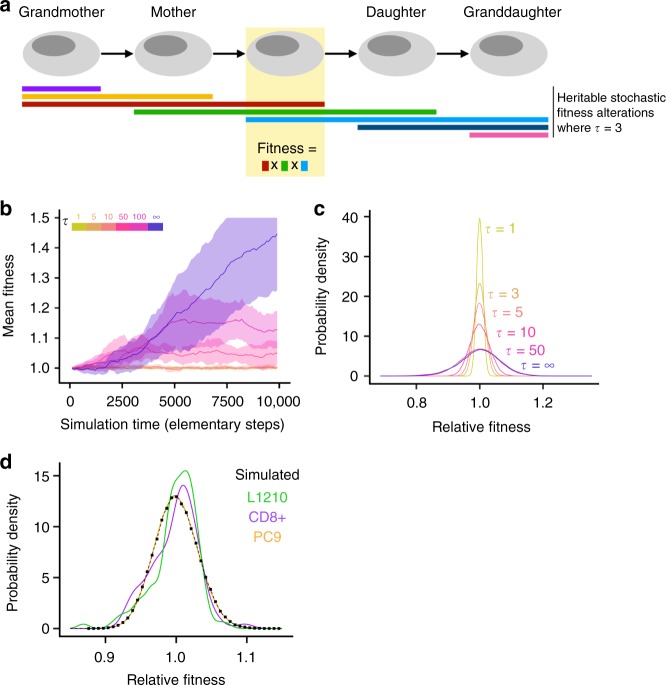
An evolutionary model incorporating non-genetic fitness changes can generate steady-state fitness variation similar to that seen in isogenic cell populations. **a** Description of the mathematical model. During a cell’s *i*th division, it acquires an epigenetic alteration (colored rectangles) that changes its fitness by effect size *m*_*i*_. This alteration is carried through the cell’s next *τ* −1 divisions. **b** Mean fitness of the population reaches a steady-state level for finite fitness effect lifetimes *τ* but diverges when non-genetic fitness changes persist indefinitely (*τ* = ∞). Shaded regions are one simulated standard deviation above and below the mean fitness at each point in simulation time (*n* = 10 independent simulations). **c** Effect of *τ* on the simulated steady-state fitness distribution of individuals of a single evolutionary stage. Increasing *τ* increases the width of the fitness distribution. Distributions are estimated from 1000 independent simulations of *N* = 100 individuals after 3600 generations. **d** Comparison of the simulated steady-state fitness distribution of a population with experimentally derived fitness distributions. The black simulated distribution with fitness variation that persists over 10 generations (*τ* = 10) is similar to properly scaled and shifted versions of the empirically estimated fitness distributions (orange: PC9/log-normal, purple: CD8+, green: L1210). The differences between the simulated distribution and the experimental CD8+ distribution (*p* = 0.99, *Z* = −0.0066) and between the simulated distribution and the experimental L1210 distribution (*p* *=* 0.99, *Z* *=* 0.0089) are not statistically significant. Simulated distributions are combined data from 1000 separate simulations (100,000 total individuals) after 3600 generations. Simulation parameter values in this figure not otherwise specified are the standard values given in Table 1

To investigate the role of background fitness variation on the dynamics of valley crossing, we considered a modified Moran process^26^ in which asexually reproducing individuals acquire (epi)genetic alterations that change their fitness during every reproductive event (Fig. 2a, Methods). During its *i*th reproductive event, an individual acquires a new multiplicative fitness effect *m*_*i*_ ~ *F*(1, *V*) drawn from a distribution of fitness effects centered around neutrality (*m*_*i*_ = 1) with variance *V*. Each stochastic fitness alteration persists through exactly *τ* cell divisions, including the division in which the alteration was acquired. If fitness effects are permanent (*τ* = ∞), the mean fitness of the population increases without bound due to selection; otherwise, the mean population fitness fluctuates around a steady-state level (Fig. 2b). The width of the steady-state relative fitness distribution increases with the variance of the fitness effect distribution, *V* (Supplementary Figure 1) and with the persistence timescale *τ* of fitness alterations (Fig. 2c). Additionally, as fitness alterations become more persistent, i.e., as *τ* increases, the shape of the steady-state fitness distribution becomes less sensitive to the shape of the fitness effect distribution, *F* (Supplementary Figure 2a-c). We found that four different fitness effect distributions *F* (log-normal, gamma, exponential, and centered Bernoulli) resulted in steady-state fitness distributions that are similar to each other (Methods, Supplementary Figure 2d). We used a log-normal distribution for *F* for the remainder of our investigations as a representative example.

Using this model, we found that the fitness distributions in simulated populations with non-genetic fitness variation were similar to fitness distributions measured in isogenic single cell experiments (Fig. 2d). Using single-cell interdivision time data, we estimated the fitness distribution of three types of cells: a human non-small cell lung cancer cell line (PC9)^27^, primary murine CD8+ T cells, and a murine lymphocytic leukemia cell line (L1210)^4^ (Methods). We found that these experimentally derived distributions were not significantly different from our simulated distributions with fitness effects that persist over 10 generations (*p* = 0.99 for both CD8+ and L1210 fitness distributions; Methods). Results from our variable fitness model are, therefore, consistent with the experimentally observed fitness distributions. However, multiple different sets of simulation parameter values (*τ, F*, and *V*) result in similar population fitness distributions. Therefore, we were unable to infer unique combinations of *τ, F*, and *V* that would best match the experimental data, but some of these parameter values are more likely to be biologically relevant than others, such as *τ* on the order of 3–20 generations^7,8^.

### Stable fitness variation promotes valley crossing

**Fig. 3:**
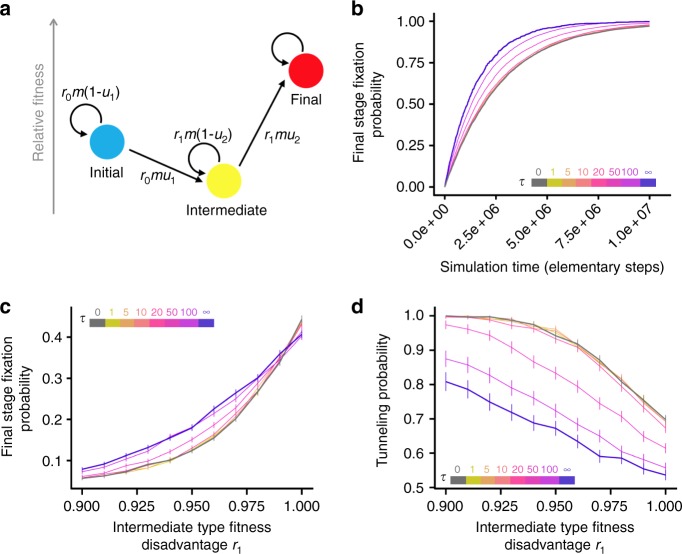
Persistent variation in fitness increases the rate of valley crossing by increasing the rate of intermediate stage fixation. **a** The definition of a fitness valley in the evolutionary model. Transition rates shown in the diagram are defined for an individual with a total stochastic fitness effect *m*. **b** Rate of valley crossing for populations with fitness alterations with different lifetimes *τ*. Less time is required for the final evolutionary stage to fix in the population as *τ* increases. Here, 10,000 simulations per model were run. **c** Simulated probability of crossing the fitness valley and reaching fixation of the final, advantageous evolutionary stage within 3650 generations across different valley depths *r*_1_. A cell generation is *N* (the population size, here 100 individuals) elementary steps; one elementary step consists of one cell dying and a new cell being born to replace it. 10,000 simulations per data point. Vertical bars are 95% confidence intervals. Line colors represent values of *τ*, as in **b**. **d** Tunneling probability across different valley depths *r*_1_. The tunneling probability is calculated as the conditional probability that tunneling occurs, given that the final advantageous stage fixes during the time of the simulation. 10,000 simulations per data point; vertical bars are 95% confidence intervals. Simulation parameter values in this figure not otherwise specified are the standard values given in Table 1

To investigate how populations with non-genetic fitness variation cross rugged fitness landscapes, we specified a two-step evolutionary model defining a fitness valley (Fig. 3a, Methods). During each reproduction event, individuals draw new random fitness effects as described above, but also may mutate to the next evolutionary stage. The population begins in the initial evolutionary stage S_0_ with average fitness *r*_0_. Here, we specify *r*_0_ = 1 for simplicity. Individuals of stage S_0_ can acquire a mutation to transition to the intermediate stage S_1_ with probability *u*_1_ during each reproductive event. This transition to the intermediate stage is associated with a multiplicative fitness cost *r*_1_/*r*_0_ < 1. Thus an S_0_ individual with fitness *w* after the stochastic fitness alteration process gives rise to a mutated S_1_ daughter with fitness *w* × (*r*_1_/*r*_0_). Intermediate-stage individuals can then mutate to the final, advantageous evolutionary stage with probability *u*_2_ per reproductive event. The fitness of the final-stage relative to that of the initial S_0_ individuals is given by *r*_2_ > *r*_0_, and the fitness benefit conferred by the transition from S_1_ to S_2_ is therefore *r*_2_/*r*_1_ > 1. We implemented this model as a stochastic computer simulation to determine the rate of crossing fitness valleys and of stochastic tunneling in populations with intra-stage fitness variability.

We found that populations with fitness variation that is stable over multiple generations cross fitness valleys more quickly than populations with no persistent fitness variation (Fig. 3b). In our model, we assume that there is no back mutation since many mutations that affect fitness are loss-of-function mutations which have a very low reversal rate. Therefore, the final evolutionary stage will eventually reach fixation in all populations. However, the state of populations at intermediate timescales differ depending on their non-genetic fitness variation properties. Here, we focused on the state of populations after 3650 generations, corresponding to timescales relevant for tumorigenesis^28^, and found that this increased rate of adaptation strongly depends on the fitness effect persistence timescale *τ* (Fig. 3c). Using the parameter values shown in Table 1, if stochastic fitness alterations only persist for approximately 1–10 generations, intra-stage fitness variation does not result in valley crossing rates that are different from those observed in the zero-variance model with no intra-stage fitness variation. Additionally, populations with stable fitness variation that acquire the final, advantageous trait are more likely to cross the fitness valley without tunneling (Fig. 3d). These observations suggest that individuals with the disadvantageous intermediate trait are more likely to fix when there is persistent fitness variation in the population, which represents a new, tunneling-independent mechanism of valley crossing.

**Table 1:** Simulation parameter values

Parameter	Value	Description
*N*	100	Number of individuals
*F*	Log-normal	Fitness effect distribution
*V*	1 × 10^−4^	Variance of *F*
*u*_1_	1 × 10^−4^	Initial to intermediate-stage mutation rate
*u*_2_	2 × 10^−4^	Intermediate to final-stage mutation rate
*r*_0_	1	Initial stage starting mean fitness
*r*_1_	0.95	Intermediate stage fitness disadvantage
*r*_2_	5	Final-stage fitness advantage
*T*_max_	3650(*N*)	Truncation time (in elementary steps)

The definitions and default values chosen for each parameter of our model. All simulations were performed with these values unless otherwise specified

Our results are robust to the shape of the fitness effect distribution (Supplementary Figure 3) and the mutation rates between the evolutionary stages (Supplementary Figure 4). The mean relative fitness advantage of the final stage affects valley crossing trajectories for lower values of the fitness advantage *r*_2_, for which drift plays a more important role in the fixation of the final evolutionary stage (Supplementary Figure 5). The effect of persistent variation is most pronounced in intermediately sized populations (Supplementary Figure 6). In large populations (thousands of cells or more), stochastic tunneling is inevitably the fastest trajectory for crossing the fitness valley. In smaller populations, drift dominates and reduces the effects of selection, leading to frequent fixation of the intermediate stage and reducing the incidence of tunneling. Finally, this increase in the valley crossing rate is observed in populations in which non-genetic fitness effects do not have fixed lifetimes, but rather have a fixed probability of reversion at every reproductive event, leading to geometrically distributed effect lifetimes (Supplementary Figure 7, Methods). In this model, even fitness effects that are expected to persist only for a few generations (>3 generations) have an impact on valley crossing dynamics, which is in contrast to our results from the deterministic lifetime model (Fig. 3c). This difference between the deterministic and geometrically distributed non-genetic effect lifetime models is likely due to very long-lived fitness effects from the heavy tail of the geometric lifetime distribution that persist beyond the mean lifetime *τ*.

### Fitness effect parameters affect valley crossing dynamics

We hypothesized that an association of the disadvantageous trait with a high-fitness background could explain the increase in the fixation probability of the intermediate stage observed in populations with fitness variation. If the disadvantageous trait arises in a particularly fit individual in the initial population that has accumulated stable beneficial fitness alterations, it will pass on that advantage to its descendants, increasing the chance of fixation of that lineage. If associations arising by chance between beneficial fitness backgrounds and the disadvantageous trait explain our results, there should be two important characteristics that affect the incidence of this novel valley crossing mechanism in a population with fitness variation: First, the frequency of emergence of intermediate-stage individuals with high-fitness backgrounds; and second, the efficacy with which these individuals pass their fitness advantage on to their descendants.

**Fig. 4:**
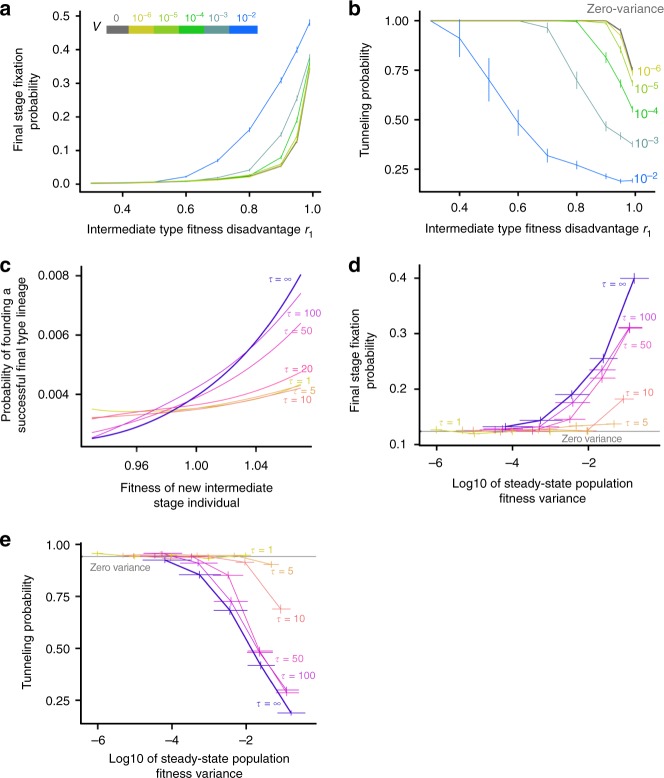
Populations with epigenetic fitness alterations that are larger or persist over more generations can cross deeper fitness valleys. **a** Probability that the final advantageous stage will fix after 3650 generations with different variances for the fitness effect distributions *V*. Different colors represent different values of *V*, with the same color mapping marked for the traces in **b**. **b** Tunneling probability increases for larger values of *V* (values marked to the right of each line) for equivalent values of the valley depth *r*_1_. 10,000 simulations per data point; vertical bars are 95% confidence intervals. **c** Smoothed probability that a new intermediate-stage individual gives rise to a final-stage individual that eventually fixes in the population. When fitness changes persist over more generations (higher values of *τ*), intermediate individuals that arise with a higher relative fitness are more likely to establish a lineage that will eventually fix. Details on how this conditional probability was estimated are given in Methods. **d** Populations with more stable fitness effects are more likely to cross fitness valleys, even if they have the same steady-state fitness variance. **e** The decrease in the tunneling probability observed for populations with higher *τ* is not dependent on the increase in steady-state distribution width associated with more stable fitness effects. Error bars are 95% confidence intervals. 10,000 simulations per point. Steady-state fitness distribution variances were estimated from 1000 independently simulated distributions. Simulation parameter values in this figure not otherwise specified are the standard values given in Table 1

The ability of a population to generate new intermediate-stage individuals with relatively high fitnesses depends on the variance *V* of the distribution of fitness effects. Larger values of *V* lead to greater variability in the fitness of new intermediate-stage mutants, in part by increasing the steady-state population variance. Increasing *V* will therefore increase the chance of generating particularly high-fitness intermediate individuals, ultimately leading to higher rates of valley crossing without stochastic tunneling. This prediction is validated by our simulation data (Fig. 4a, b, Supplementary Figure 8). We also found that populations with larger values of *V* are able to cross deeper fitness valleys, again because they are able to produce a small number of intermediates with relatively high fitnesses despite large fitness disadvantages due to the deleterious mutation.

The fitness effect persistence lifetime *τ* influences the fixation probability of intermediate-stage individuals by modulating the effect of a beneficial fitness background of a new intermediate individual. Lower values of *τ* (less persistent non-genetic fitness alterations) imply that any background fitness gains present in a particular intermediate-stage individual will not be passed down to many of its descendants and will not persist over the long timescales needed to substantially affect its overall fixation probability. Indeed, we found that for lower values of *τ*, intermediate individuals that initially have high relative fitness are about as likely to generate a successful lineage as individuals with lower initial fitness (Fig. 4c). However, as *τ* increases, intermediate-stage individuals with higher relative fitnesses become more likely to establish a successful final-stage lineage. The beneficial fitness alterations present in these intermediate individuals are able to persist through several reproductive events and ultimately have a greater impact on the fate of the new lineage. The stability of the fitness effects across multiple generations remains an important determinant of valley crossing trajectories even after controlling for the increase in the steady-state fitness distribution width at higher values of *τ* (Fig. 4d, e).

## Discussion

Here, we characterized a mathematical model describing the rate and mechanisms of adaptation to rugged fitness landscapes of populations with inter-individual variation in fitness. Using this model, we showed that populations with fitness variation that persists across multiple generations can cross fitness valleys more quickly than populations without such variation; the evolutionary mechanism by which these populations adapt to such landscapes allows them to fix the deleterious intermediate stage by hitchhiking on stochastically accumulated high-fitness backgrounds. The magnitude of this effect strongly depends on the number of generations over which fitness variation persists, the valley depth, and the variance of the fitness effect distribution. Our analyses revealed that populations with frequent fitness alterations mediated by epigenetic mechanisms that are stable over multiple generations are more likely to cross fitness valleys without stochastic tunneling.

The dependence of this effect on the fitness effect lifetime is supported by previous work on similar evolutionary phenomena. Prior characterizations of the Hill–Robertson effect emphasize that recombination reduces the impact of a variable fitness background on the fate of a linked mutation^9,10^. In those contributions, the recombination rate effects are related to the timescale of association of the fitness background with the genetic mutations that specify the fitness valley—more recombination implies that the fitness background has less of an impact on the fate of a mutation over the time period during which it is segregating in the population. Therefore, if the linkage between fitness background and the trait in question is weak, theoretical descriptions of Hill–Robertson interference predict that the effect of fitness variation on selection is minimal. Similar observations were made in our model when *τ* was small. Note that the effect of the fitness effect lifetime *τ* on the adaptation rate is modulated by other parameters in our model such as the amount of standing fitness variation in the population. Therefore, there is no constant critical threshold of *τ* for all populations below which stochastic fitness alterations have a negligible impact on evolution.

The depth of fitness valleys that can be crossed with our observed evolutionary mechanism increases with enhanced fitness variation. Many deleterious mutations have low selection coefficients (corresponding to valley depths *r*_1_/*r*_0_ *>* 0.9) as determined experimentally^1,29^, corresponding to the parameter regime in which we observe this effect. Therefore, our proposed mechanism for crossing fitness valleys may affect all evolutionary trajectories through these deleterious states even at minimal fitness distribution variances (*V* ~ 10^−4^–10^−5^) by increasing the rate of fixation of deleterious mutations.

The population size *N* is particularly important for determining drift-selection dynamics relevant to valley crossing situations. Previous work has demonstrated that intermediately sized populations are governed by ‘one-hit’ dynamics when crossing fitness valleys^19^. By accelerating the rate-limiting step of generating a substantially sized population of intermediate-stage individuals, persistent fitness variation speeds up adaptation in this range of population sizes. Such situations are of special interest in carcinogenesis; for instance, colonic crypts are populated by a small number of stem cells^30^. In such situations, increasing the amount of fitness variation (for example by acquiring a mutator phenotype) may increase the rate of fixing mutations with mildly deleterious fitness effects that can nevertheless lead to further adaptation.

While we demonstrated that stable fitness variation increases the rate of adaptation of a population in a specific fitness landscape topology (fitness valleys), this type of heterogeneity also has important consequences that affect evolution through other fitness landscapes. One immediate consequence of the observed increased fixation probability of weakly deleterious mutations is an increased deleterious mutational load. These deleterious mutations may potentiate further adaptation by acting as intermediate stages in fitness valleys but may also lower the overall population fitness. Furthermore, just as background fitness variation reduces the strength of selection against deleterious mutations, it also reduces the strength of selection for weakly beneficial mutations, possibly hindering adaptation. These effects may influence the way heterogeneous populations move through fitness landscapes. Therefore, further theoretical and experimental studies on more general fitness landscapes are warranted and will lead to additional insight into the consequences of non-genetic sources of inter-individual fitness variation.

## Methods

### Computational evolutionary model with fitness variation

Our model is a variation of the standard Moran model of evolutionary dynamics that describes fitness valley crossing with different amounts of fitness variation that persist over one or more generations. In the Moran model, S_0_, S_1_, and S_2_ denote the initial, intermediate, and final evolutionary stages (or ‘types’ of individuals), respectively. The process initiates with a population of *N* asexually reproducing S_0_ individuals. At every elementary time step, one individual is chosen to reproduce randomly with a probability that is proportional to its fitness. Another individual is chosen at random to die. The reproducing individual (‘mother’) gives rise to two daughters; one daughter replaces the mother, and the other replaces the dead individual, so that the total population size remains constant at all times. When dividing, one daughter may mutate to the next evolutionary stage with probability *u*_1_ if the mother is S_0_ and *u*_2_ if the mother is S_1_. No back mutation is permitted. The process continues until S_2_ fixation is achieved or for *T*_max_ elementary steps, whichever comes first.

Here, we refer to the simplest model where all individuals of the same evolutionary stage have the same fitness as the “zero-variance” model. We compared this zero-variance model with a Moran model that incorporates fitness variation within evolutionary stages. In this new model, fitness variation within stages is caused by stochastic alterations in fitness that occur with every reproductive event. These alterations are described by a distribution of fitness effects that defines the probability of acquiring an alteration with a given fitness effect, and an effect lifetime *τ* that specifies the number of generations for which each alteration is inherited.

More specifically, let a particular individual in a population have absolute fitness *w*_*i*_ after its *i*th reproduction. This fitness

$$w_i = r_j\mathop {\prod }\limits_{k = i - \tau + 1}^i m_k$$

is the product of an evolutionary stage-specific fitness effect *r*_*j*_ for a stage-*j* individual and one or more stochastically acquired fitness alterations *m*_*k*_. The values *r*_*j*_ (which have the value *r*_*0*_ for initial-stage individuals, *r*_1_ for intermediate-stage individuals, and *r*_*2*_ for final-stage individuals; *r*_2_ > *r*_0_ > *r*_1_) define the depth of the fitness valley. For simplicity, *r*_0_ is set to 1 for the entire study. The fitness alterations *m*_*k*_ are drawn at each reproductive event *k* from a fitness effect distribution *F* with mean 1 and variance *V*. Mothers pass on the same value of the fitness alteration *m*_*k*_ to both of their daughters at reproduction *k* to recapitulate the high interdivision time correlations between sister cells observed in single cell experiments^3^. Each fitness alteration persists through exactly *τ* reproductive events, including the reproductive event that generated that alteration. Therefore, when *τ* = 1, fitness alterations are not passed down to subsequent generations. In this case, during each reproduction event, a new fitness for the dividing cell is selected according to *F*, and the steady-state relative fitness distribution of a population of a single evolutionary is equivalent to *F*. Conversely, as *τ* goes to infinity, fitness alterations are permanent. The zero-variance model both represents the limit as *τ* goes to 0 and the limit as *V* goes to 0.

All fitness modifications are modeled on the multiplicative scale (i.e., as a percent change in fitness, rather than an addition or subtraction) to ensure that the relative fitness advantage or penalty associated with a mutation does not change as the absolute population mean fitness drifts upward in populations with long *τ*. However, our results also hold when the fitness modifications are modeled on an additive scale (Supplementary Figure 9); the disadvantage of this approach is that the fitness penalties and bonuses associated with a mutation diminish as the overall fitness of the population grows.

In most of our simulations, all sources of non-genetic fitness changes that occur during a single reproduction create a single fitness alteration with one combined fitness effect and a deterministic lifetime. However, in Supplementary Figure 7, each non-genetic fitness alteration that occurs during reproduction is modeled as a separate event. In this modified version of the model, a Poisson-distributed number of these events occurs during reproduction, with an average of one event occurring per reproduction. During subsequent generations, each individual alteration has a fixed probability of reversion, leading to fitness effect lifetimes that are geometrically distributed. Simulations of this evolutionary model are parameterized by the expected fitness effect lifetime *τ*.

### Fitness effect distributions

We investigated four different distributions for *F*: the log-normal, double-exponential (Laplace), gamma, and centered Bernoulli distributions. Parameters for these distributions were chosen to provide mean 1 and the appropriate variance. The log-normal distribution was used for the majority of simulations, since the steady-state population fitness distribution is similar to the experimentally measured distributions, even when fitness effects do not persist beyond a single generation. The log-normal distribution provides similar results to simulations using the other distributions, including the heavier-tailed exponential and gamma distributions, which have previously been shown to describe mutational fitness effect distributions^31,32^. The centered Bernoulli distribution represents the case in which all epigenetic changes have the same fitness effect magnitude and are beneficial or deleterious with the same probability, and is defined by the probability mass function

$$P\left( k \right) = \left\{ {\begin{array}{*{20}{c}} {\frac{1}{2}{\mathrm{for}}\,k = - \sqrt V } \\ {\frac{1}{2}{\mathrm{for}}\,k = \sqrt V } \end{array}} \right..$$

### Estimation of single cell fitness distributions

The CD8+ and L1210 fitness distributions were estimated from single cell interdivision time data for two types of murine cells^4^ (Fig. 1b). For the PC9 cell line, we leveraged previous work^33^ demonstrating that an exponentially modified Gaussian provides a good fit to the intermitotic time distribution of these cells. We therefore used the maximum likelihood parameter estimates for PC9 cells treated with dimethyl sulfoxide (DMSO) reported previously to estimate the PC9 fitness distribution^27^ (Supplementary Figure 10). To estimate the corresponding fitness distributions for all three cell types, we considered fitness to be inversely proportional to interdivision time; this assumption enables us to create an empirical fitness distribution for each cell type.

### Comparison of simulated/experimental fitness distributions

To determine whether the experimentally measured CD8+ and L1210 single cell fitness distributions could have been generated by our evolutionary model with permanent non-genetic fitness alterations, we estimated the probability that the experimentally measured distributions would be at least as different from our simulated fitness distributions as we observed, using a two-tailed goodness-of-fit *z*-test^34^. Specifically, we tested the null hypothesis that

$$H_0:g\left( x \right) = h(x),$$

where *g*(*x*) is the experimentally measured fitness distribution (CD8+ or L1210) and *h*(*x*) is the simulated fitness distribution of our model, with a log-normal fitness effect distribution and non-genetic fitness effects that persist over 10 generations (*τ* = 10). The experimental distribution is sampled by *n* i.i.d. measured cell fitnesses *X*_*j*_, which are scaled and shifted to have the same mean and variance as the simulated distribution. The mean-squared error (MSE) between the distributions

$$\begin{array}{ccccc}\\ {\rm MSE} = & {\int} {\left( {g\left( x \right) - h(x)} \right)^2{\rm d}x} \\ \\ & = {\int} {g^2\left( x \right){\rm d}x + } {\int} {h^2\left( x \right){\rm d}x - 2} {\int} {g\left( x \right)h\left( x \right){\rm d}x} \\ \end{array}$$

can be estimated as

$$\widehat {{\rm MSE}} = \frac{1}{n}\mathop {\sum }\limits_{i = 1}^n \widehat {g_{ - i}}\left( {X_i} \right) + {\int} {\hat h^2\left( x \right){\rm d}x - \frac{2}{n}} \mathop {\sum }\limits_{i = 1}^n \hat h(X_i),$$

where $\widehat {g_{ - i}}(x)$ is the leave-one-out Gaussian kernel density estimate of *g*(*x*) from all experimental data except the measurement *X*_*i*_ and $\hat h(x)$ is the Gaussian kernel density estimate of the simulated distribution *h*(*x*). This estimated MSE is asymptotically normal under the null hypothesis, with the test statistic

$$\frac{{\widehat {{\rm MSE}}}}{{\left( {\mathop {\sum }\nolimits_{i = 1}^n \mathop {\sum }\nolimits_{j = 1}^n K^2\left( {\frac{{X_i - X_j}}{h}} \right)} \right)^{1/2}}}\mathop { \to }\limits^D N(0,1),$$

where *K* is the Gaussian kernel function with bandwidth *h* used to estimate *g*(*x*) above.

### Estimation of simulated tunneling and fixation probabilities

Our results focus on differences in the estimated final-stage fixation probability (i.e., proportion of simulations that result in fixation of final stage individuals before 3650 generations have passed) and the tunneling probability between simulated conditions. Here, we define the tunneling probability as the conditional probability that intermediate stage individuals fix at some point during the simulation, given that the final stage fixes by the end of the simulation. We estimated the tunneling probability as

$$\hat P({\mathrm{tunnel}}|{\mathrm{fix}}) = \frac{\hat P({\mathrm{tunnel}}|{\mathrm{fix}})}{{\hat P{\mathrm{(fix)}}}}$$

where the joint probability of tunneling and final stage fixation and the fixation probability are directly estimated from the proportion of simulations with final-stage fixation and tunneling or the frequency of final-stage fixation, respectively.

We used a similar strategy to estimate the conditional probability that a new intermediate-stage mutant will give rise to a final stage lineage that fixes by the end of the simulation, given that it arises with a specific relative fitness. Using Bayes’ theorem, we estimated

$$\hat P({\mathrm{fix}}\,{\mathrm{final}}\,{\mathrm{stage}}|{\mathrm{fitness}}) = \frac{{\hat P{\mathrm{(fitness}}|{\mathrm{fix}}\,{\mathrm{final}}\,{\mathrm{stage)}}\hat P{\mathrm{(fix}}\,{\mathrm{final}}\,{\mathrm{stage)}}}}{{\hat P{\mathrm{(fitness)}}}}$$

by estimating the density of fitnesses of new intermediate-stage individuals that eventually lead to final-stage fixation (the conditional distribution *P*(fitness|fix final stage)), the total density of all new intermediate stage individuals *P*(fitness), and the overall frequency of new intermediate stage individuals that eventually lead to final-stage fixation. In Fig. 4c, the fitness distributions for each condition were estimated from all intermediate individuals created by de novo mutation in 10,000 independent simulations (approximately 300,000 individuals total). Probability density functions were estimated by Gaussian kernel smoothing in R (version 1.0.143). The total probability of fixation of new intermediate-stage individuals was estimated as the proportion of all new intermediate individuals that generate a successful final stage lineage in 10,000 independent simulations per condition.

### Study design and reproducibility

The number of simulations performed for each condition (typically 10,000 trials) was chosen such that enough adaptation events were observed to confidently estimate the mean tunneling probability for each condition. For a conservative estimate of a final-stage adaptation probability of 0.1, approximately 1000 adaptation events were observed, leading to an acceptable standard error of the estimated tunneling probability of at most 0.016 (CV ~2%).

The findings from this study were verified for limiting cases of the simulation parameters with separate custom simulation code by one of the authors who had not seen the software used to generate the data presented in the paper. None of the conditions tested had results that were unable to be reproduced. No data were excluded from analysis. As this was a theoretical study, no blinding or randomization methods were used.

### Code availability

The stochastic evolutionary dynamics C++ software package used for all simulations is available on GitHub (https://github.com/Michorlab/evo_sim), with documentation. Input files to the simulation software used to generate the results presented here are available at https://doi.org/10.7910/DVN/5D6YPB within each individual data directory. Custom Python 2.7 and R scripts (R version 3.4.1) used for data analysis and visualization are available with the data at https://doi.org/10.7910/DVN/5D6YPB.

## Electronic supplementary material

Supp Material

## Data Availability

Raw simulation data generated and analyzed in this study are available in the Harvard Dataverse at https://doi.org/10.7910/DVN/5D6YPB.
