# Supplementary material for: Fitness variation in isogenic populations leads to a novel evolutionary mechanism for crossing fitness valleys
Source: Commun Biol. 2018 Sep 26;1:151. doi: 10.1038/s42003-018-0160-1 (PMC6158234; doi:10.1038/s42003-018-0160-1)
Supplement: Supplementary file 1 — Supp Material [file 42003_2018_160_MOESM1_ESM.pdf]

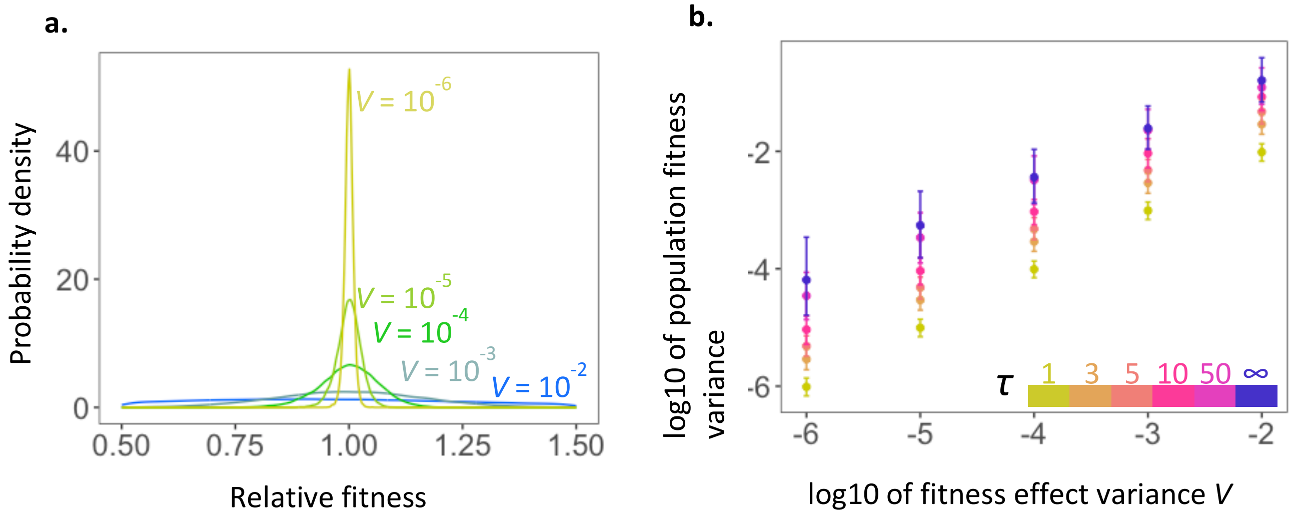

Supplementary Figure 1: Steady-state fitness distributions in the population depend on the variance *V* of the fitness effect distribution. a. Simulated fitness distributions at steady state. Our model only specifies the distribution of fitness effects, *F*, which leads to variation in the overall fitness of individuals in the population. This fitness effect distribution *F* has a specified variance *V*, which influences the fitnesses of the individuals in the population. The panel (**a**) shows the effect of *V* on the simulated steady-state fitness distribution of individuals of a single evolutionary stage. Increasing *V* increases the width of the fitness distribution at steady-state. Here, fitness alterations are permanent ($\tau = \infty$). **b.** The variance of the simulated fitness distributions (i.e. the distributions plotted in (**a**)) depends on the variance *V* of the specified fitness effect distribution *F*. As we specify greater values of *V*, increasing the magnitude of stochastic fitness alterations acquired during each reproductive event, the overall steady-state population fitness variance increases as well. Colors represent the fitness effect lifetimes τ, as shown in the color bar. Results are from 1000 independent simulations; error bars are simulated 95% confidence intervals. All panels were generated from simulations of N=100 individuals at time = 3,600 generations after initiation of the simulation. See **Table 1** for other parameter values.

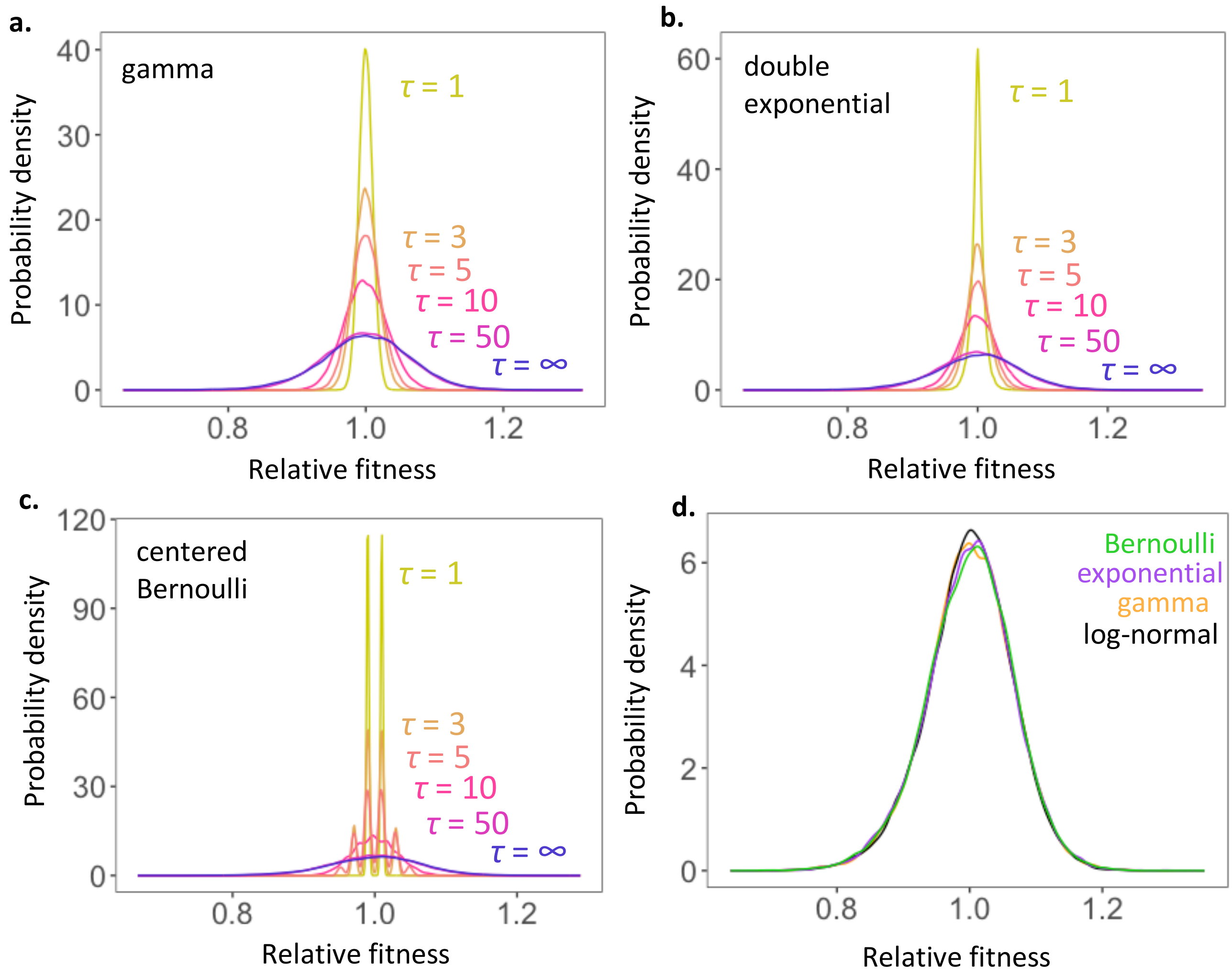

Supplementary Figure 2: The shape of simulated steady-state population fitness distributions generated by stable fitness variation is insensitive to the shape of the input distribution of fitness effects, *F*. a-c. Simulated population-level relative fitness distributions with gamma (**a**), double-exponential (**b**), and centered Bernoulli (**c**) fitness effect distributions *F*. During each reproductive event, the daughters draw a new fitness alteration from the distribution *F*. Their new fitness is equal to the mother's fitness multiplied by this stochastic fitness alteration value. Different shapes of *F* may therefore affect the properties of the population fitness distribution at steady-state. However, as the fitness effect lifetime τ increases, we found the population fitness distributions converge to a single distribution, exemplified by the results given by the permanent fitness alteration model ($\tau = \infty$). In particular, the modified Bernoulli fitness effect distribution (**Methods**) generates smoother steady-state fitness distributions when fitness effects are more stable, i.e. as τ goes to infinity. The distinctive shape and discontinuities of the fitness effect distribution have less of an impact on the overall fitness distribution as more fitness effects accumulate in the population, which occurs at higher values of τ. **d.** Steady-state population fitness distributions for $\tau = \infty$ with different fitness effect distribution shapes *F*. Different *F* shapes are denoted by different colors (labeled on top right); all fitness effect

distributions have the same variance $V = 10^{-4}$. At longer fitness effect lifetimes τ, the shape of the overall fitness distribution is insensitive to the shape of F. All simulated distributions in this figure are estimated by smoothing data combined from 1000 separate simulations of $N = 100$ individuals each (100,000 individuals total) of 3600 generations. See **Table 1** for other parameter values.

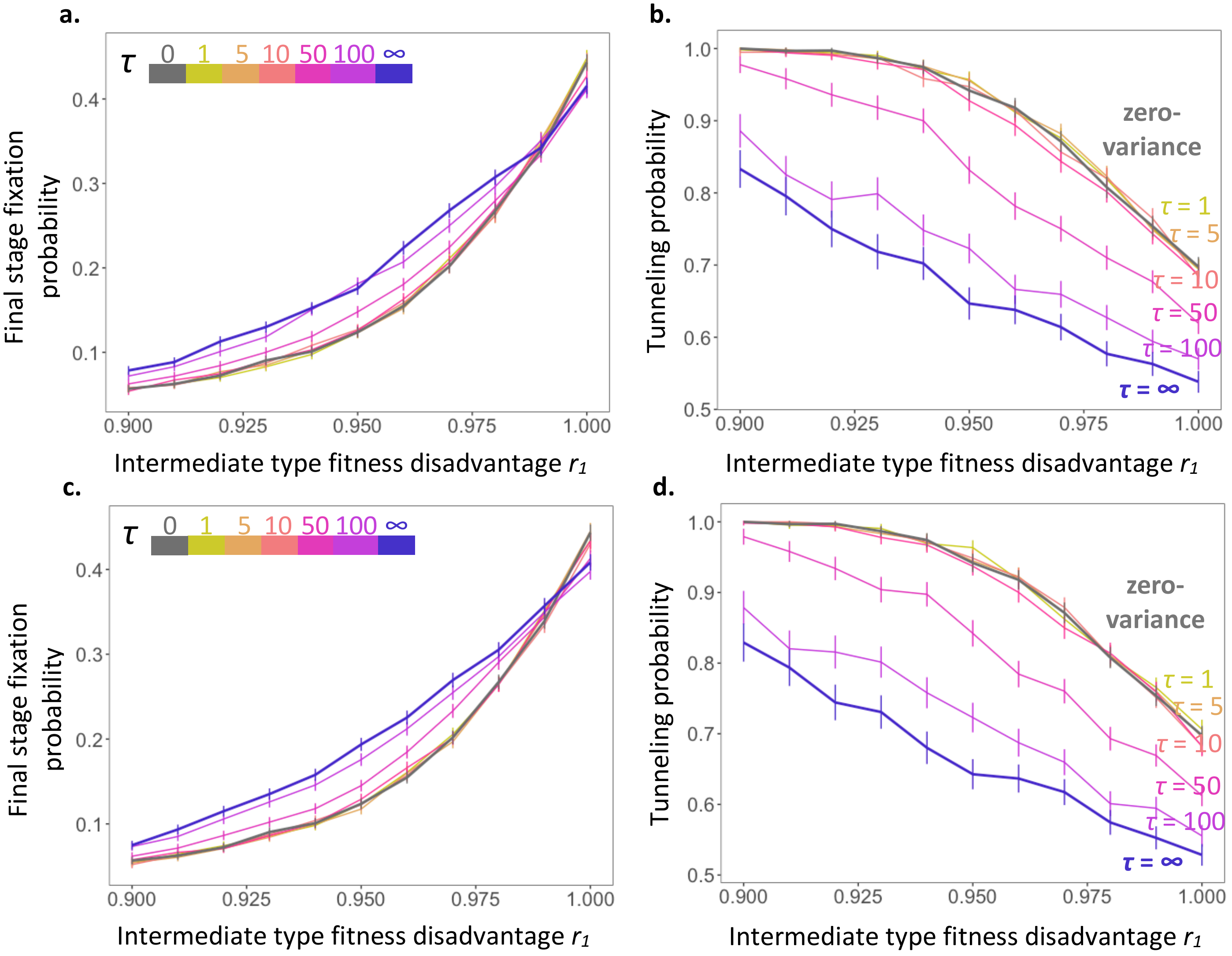

Supplementary Figure 3: Sensitivity analyses of the fitness effect distribution, *F*, demonstrate robustness in valley crossing dynamics. **a, b.** The final stage fixation probability (**a**) and the tunneling probability (**b**) of a population with a centered Bernoulli distribution of fitness effects (**Methods**) over different valley depths r_1 are similar to those presented in **Figure 4**, where a log-normal distribution was used instead. **c, d.** The final stage fixation probability (**c**) and the tunneling probability (**d**) of a population with a double exponential distribution of fitness effects (**Methods**) over different valley depths r_1 are similar to those presented in **Figure 4**, where a log-normal distribution was used instead. Colors indicate values of *τ;* the same color scheme was used in all panels. See **Table 1** for other parameter values. 10,000 simulations were run per condition, and vertical bars represent 95% confidence intervals.

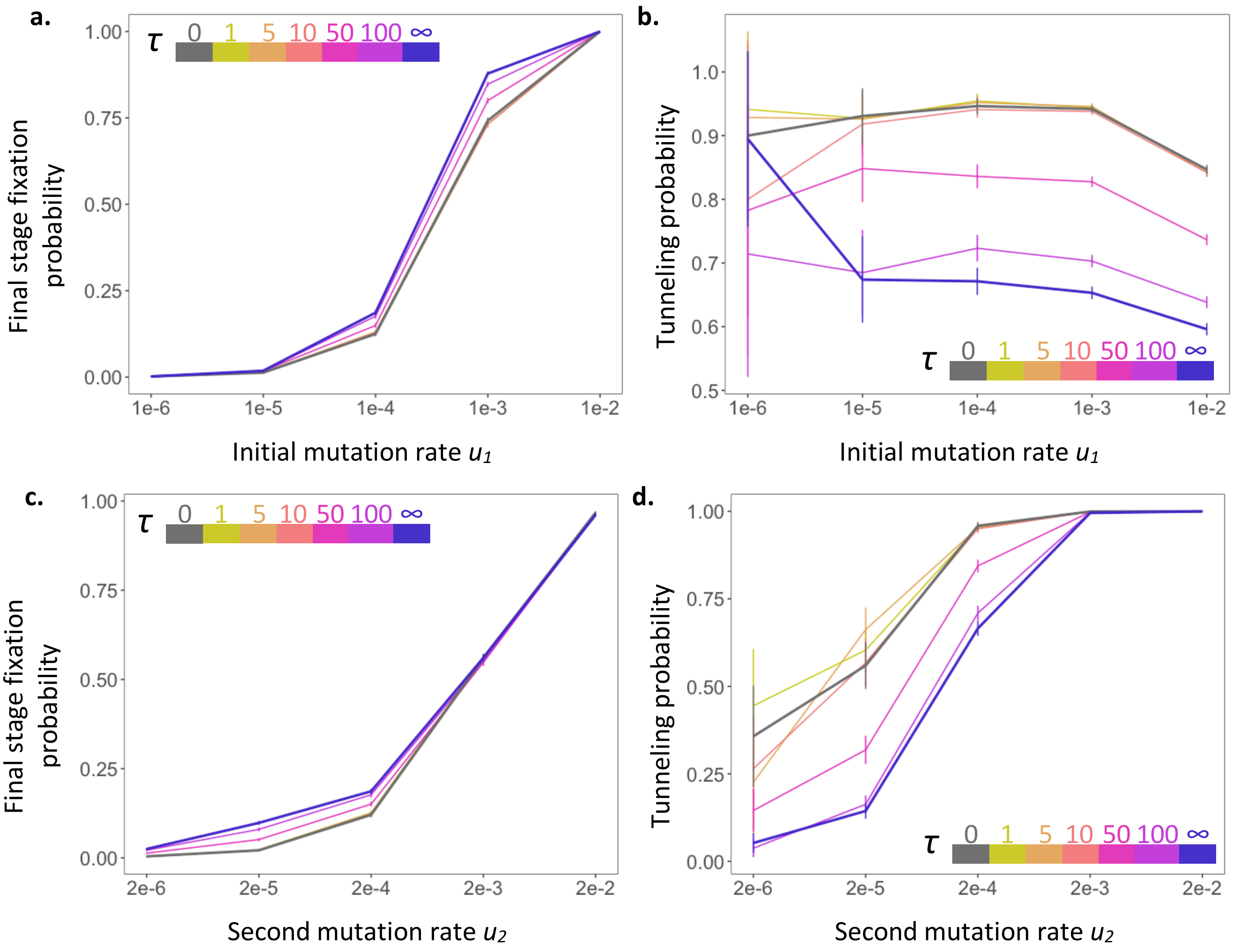

Supplementary Figure 4: Persistent fitness variation increases the rate of valley crossing over a range of mutation rates. Mutation rates were chosen to be relatively high for convenience of simulation. Increasing either the first mutation rate u_1 (**a**) or the second mutation rate u_2 (**c**) increases the rate of final stage fixation. While changing the first mutation rate u_1 has little effect on the tunneling probability (**b**), increasing the second mutation rate u_2 increases the tunneling probability (**d**). This effect arises because higher values of u_2 increase the rate of emergence of final stage individuals from new intermediate lineages, allowing the final stage to fix before the intermediate stage has taken over the population. Colors indicate values of τ; see **Table 1** for other parameter values. All panels reflect 10,000 simulations per condition. Vertical bars are 95% confidence intervals.

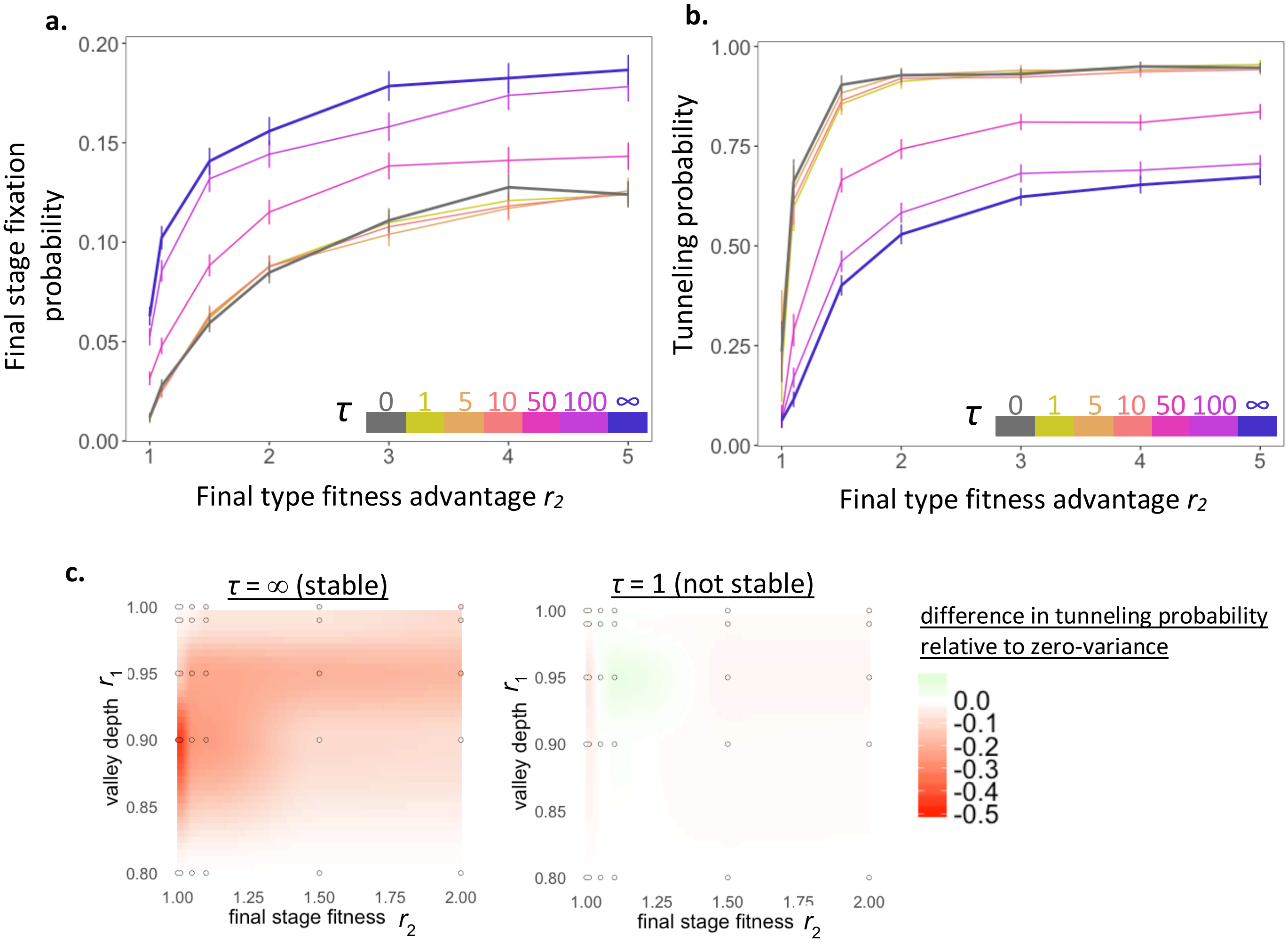

Supplementary Figure 5: Persistent fitness variation leads to a reduced reliance on stochastic tunneling over a range of final stage fitness advantages r_2. a. As expected, the adaptation probability increases with the final type fitness advantage r_2, for all values of r_2 tested. Colors represent fitness effect lifetimes τ. **b.** The tunneling probability also increases with r_2. A lower final-stage fitness r_2 decreases the rate at which final stage individuals fix, making tunneling more difficult. Stable variation leads to lower tunneling probabilities at values of r_2 that are sufficiently large to support stochastic tunneling in the zero-variance model. Colors represent the same fitness lifetime values τ as in (**a**). **c**. The effect of r_2 changes for different values of r_1 in the persistent nongenetic variation model, but almost no difference in the tunneling probability relative to the zero-variance Moran model was observed when $\tau = 1$, i.e. fitness variation does not persist through reproduction. See **Table 1** for other parameter values. 10,000 simulations per condition. Vertical bars are 95% confidence intervals.

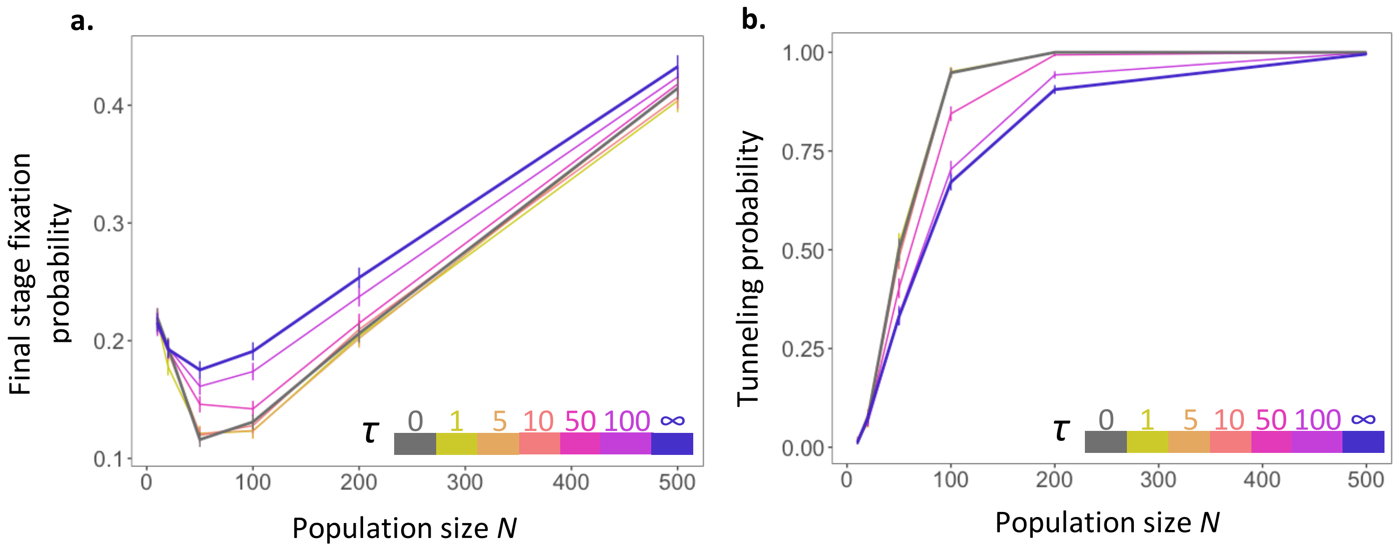

Supplementary Figure 6: Different population sizes *N* lead to different valley-crossing dynamics. a. The final stage fixation probability is higher as compared to the zero-variance model (grey) when there is stable fitness variation in the population across a range of population sizes. Different values of the fitness effect lifetime τ is denoted by the line color; see (**b**) for the color scheme. **b.** In very small populations, the dominant mechanism of valley crossing is sequential fixation, where the intermediate stage reaches fixation before individuals of the final stage emerge. In very large populations, tunneling is the dominant mechanism because the waiting time to generate an individual of the final stage is short compared to the time it takes for an individual of the intermediate stage to take over the population. See **Table 1** for other parameter values. 10,000 simulations per point. Vertical bars are 95% confidence intervals.

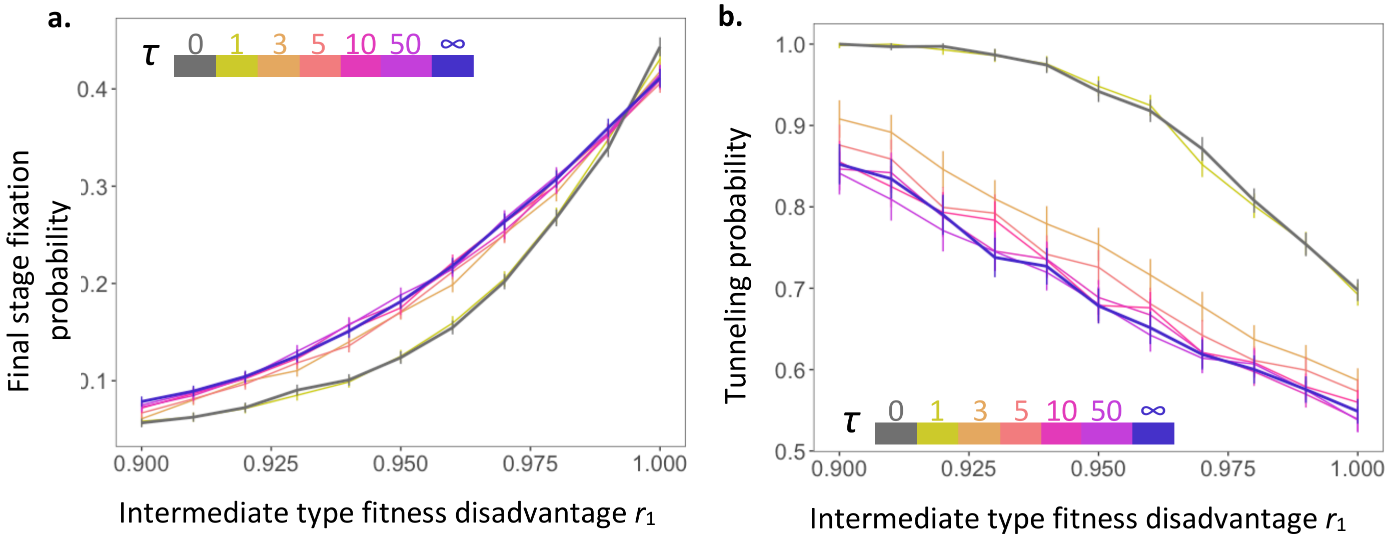

Supplementary Figure 7: Populations with geometrically-distributed nongenetic fitness effect lifetimes cross fitness valleys faster than populations with no nongenetic fitness variation. a. Here, the persistence length of nongenetic alterations is no longer deterministic, but is geometrically distributed with an expected lifetime τ. Under this model, a similar increase in the valley crossing rate is found, except that this effect is magnified at lower values of τ. **b.** The relative tunneling probability again decreases as the expected lifetime τ increases. The tunneling probability decreases more rapidly than in simulations with deterministic fitness effect lifetimes for increasing τ. See **Table 1** for other parameter values. 10,000 simulations per condition. Vertical bars are 95% confidence intervals.

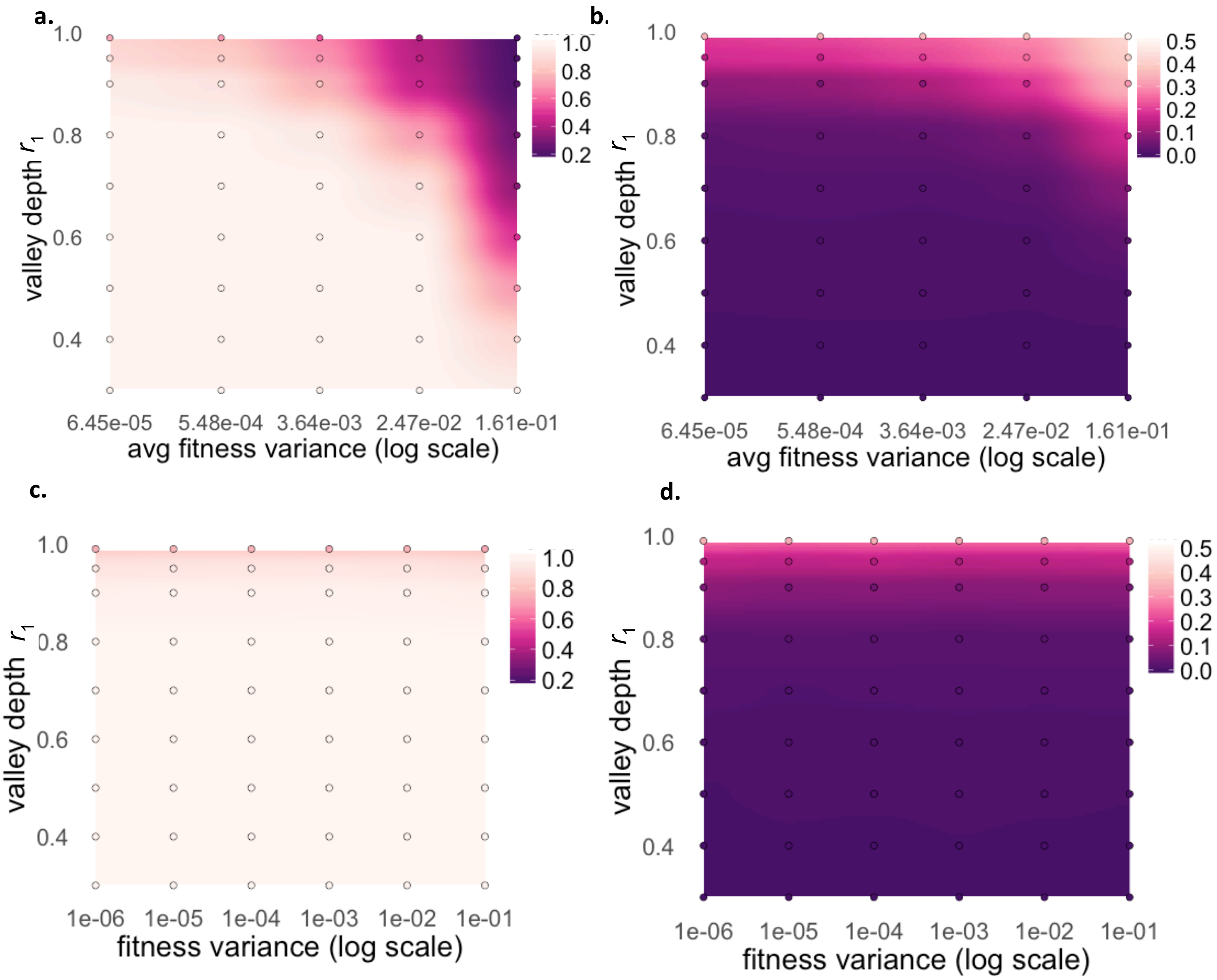

Supplementary Figure 8: Populations with greater fitness variability can cross deeper fitness valleys. a, b. Simulated tunneling probability (**a**) and adaptation probability (**b**) depending on both the relative intermediate fitness r_1 and the estimated variance of the overall fitness distribution in the model with permanent nongenetic fitness variation ($\tau = \infty$). The estimated variances on the x-axis describe the width of the overall simulated population fitness distribution, with higher estimated variances corresponding to more inter-individual variability in fitness. The estimated variances were calculated as the mean sample variance of the population fitness distribution from 1000 independent simulations, after 3600 generations. **c, d.** Simulated tunneling probability (**c**) and adaptation probability (**d**) depending on both the relative intermediate fitness r_1 and the variance of the overall fitness distribution in the model where fitness changes do not persist through reproduction ($\tau = 1$). In (**c**) and (**d**), the population fitness distribution has exactly the same variance V as the fitness effect distribution, so the fitness variance on the x-axis was not estimated by simulation. In all panels, simulated data

(circles) were interpolated to generate the entire heatmaps. Each point generated using 10,000 simulations. See **Table 1** for other parameter values.

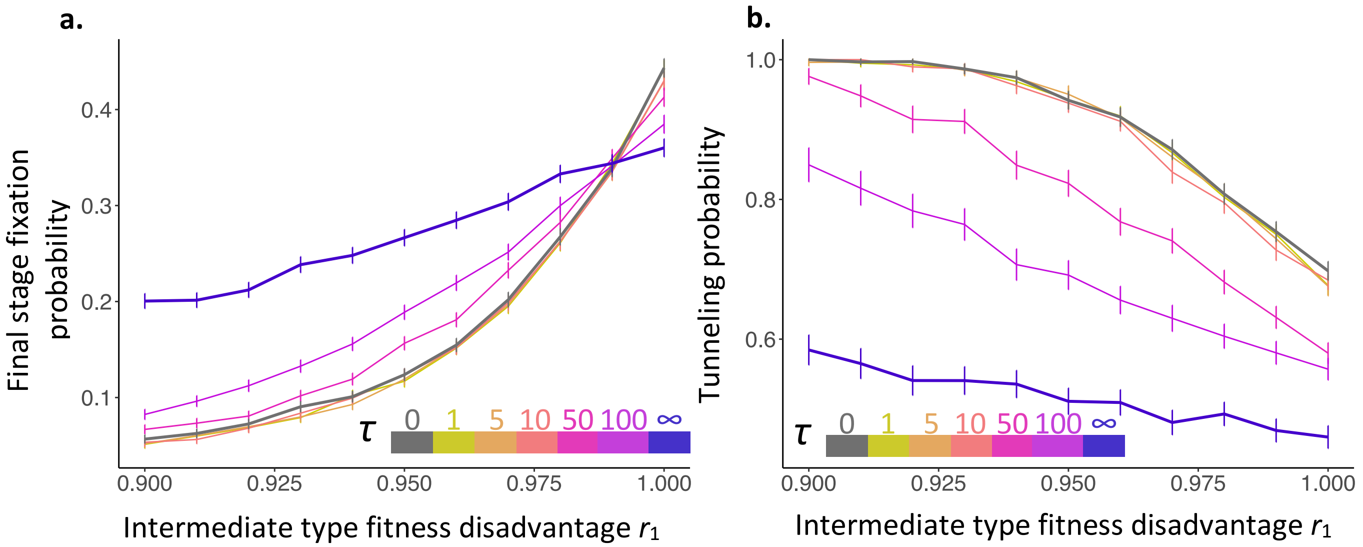

Supplementary Figure 9: Persistent stochastic fitness alterations with additive fitness effects also lead to more rapid valley-crossing. Throughout, we modeled fitness modifications from mother to daughter to occur on a multiplicative rather than additive scale to ensure that the relative penalty or bonus associated with a mutation remains constant as the fitness of the cells in the population increases. If these fitness modifications were modeled on an additive basis (i.e. if we penalized mutation by a fixed quantity, rather than a fixed percentage), the increase in adaptation rate and decrease in reliance on tunneling under the stable fitness variation model are even more pronounced. Thus our results are robust to assumptions of additivity versus multiplicativity. All parameter values are as in **Table 1**. Vertical bars are 95% confidence intervals.

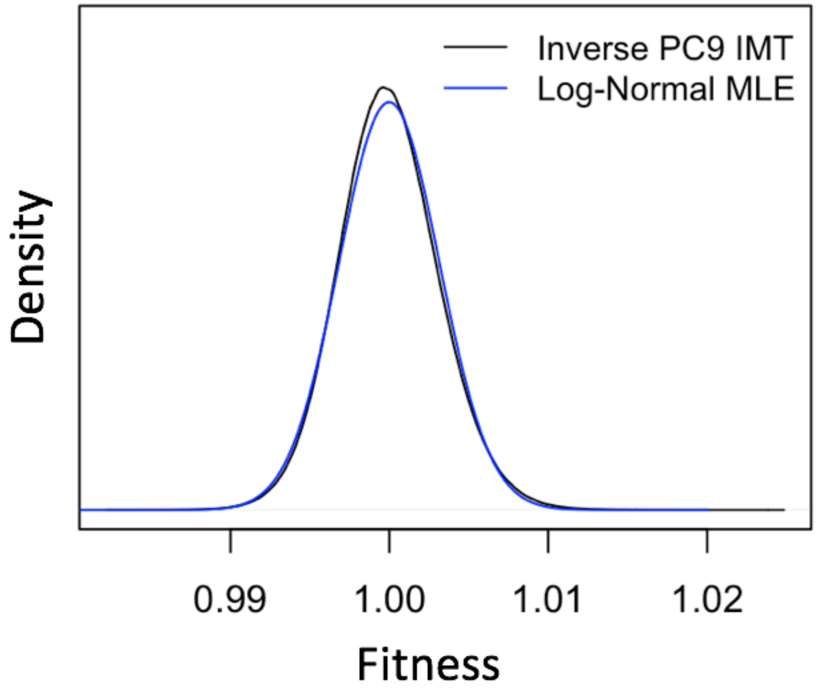

Supplementary Figure 10: Log-normal approximation to the PC9 fitness distribution. The PC9 fitness distribution was estimated by generating one million samples from the exponentially-modified Gaussian model fit to the DMSO-treated PC9 intermitotic time (IMT) data[27]. The black line represents the smoothed density of the multiplicative inverse of these samples (scaled and re-centered to have mean one and variance *V*). The blue line represents the density of the log-normal MLE of these data. Given the similarity between these two densities, we plotted the PC9 steady-state fitness distributions in **Fig. 1** and **Fig. 2** as log-normal distributions.
